# A Link Work Intervention to Facilitate Dental Visiting in People With Severe Mental Illness: A Two‐Arm, Multi‐Site, Assessor Blind, Randomised Feasibility Trial With Dental Record Linkage

**DOI:** 10.1111/cdoe.70002

**Published:** 2025-08-04

**Authors:** Jasper Palmier‐Claus, Abigail Morris, Paul French, Robert Griffiths, Vishal R. Aggarwal, Katherine Berry, Efstathia Gkioni, Rebecca Harris, Louise Laverty, Fiona Lobban, Sarah Procter, Eirian Kerry, Connie Newens, Pauline Mupinga, Rebecca Golby, Kyriakos Valemis, Lucy Oakes, Fanni Fazekas, Antonia Perry, David Shiers, Christopher Lodge, Claire Hilton, Alison Dawber, Emma Elliott, Farah Lunat, Girvan Burnside

**Affiliations:** ^1^ The Spectrum Centre for Mental Health Research, Lancaster University Lancaster UK; ^2^ Lancashire & South Cumbria NHS Foundation Trust Preston Lancashire UK; ^3^ Pennine Care NHS Foundation Trust Ashton‐under‐Lyne UK; ^4^ Manchester Metropolitan University Manchester UK; ^5^ Division of Nursing, Midwifery & Social Work University of Manchester Manchester UK; ^6^ Greater Manchester Mental Health NHS Foundation Trust Manchester UK; ^7^ School of Dentistry University of Leeds Leeds UK; ^8^ Division of Psychology & Mental Health University of Manchester Manchester UK; ^9^ Liverpool Clinical Trials Centre, Clinical Directorate University of Liverpool Liverpool UK; ^10^ Institute of Population Health, University of Liverpool Liverpool UK

**Keywords:** dentistry, dentists, mental health, mood disorders, psychosis

## Abstract

**Objectives:**

People with severe mental illness experience poor oral health, compared to the general population. They experience inequity in accessing dental services. This randomised controlled trial evaluated the acceptability and feasibility of a link work intervention to support people with severe mental illness to access a routine dental appointment.

**Methods:**

This was a feasibility randomised controlled trial across three sites with 1:1 allocation to Treatment as usual (TAU) or TAU plus a link work intervention (ISRCTN13650779). Participants were adults accessing mental health services who had not attended a routine dental appointment in the past 3 years. The intervention comprised up to six sessions with a link worker. Participants completed self‐report assessments and an optional dental examination at baseline and after nine months. Dental visiting data were obtained through self‐report and the NHS Business Services Authority (BSA).

**Results:**

One hundred and sixty‐one participants were referred into the trial, resulting in 79 out of the target 84 randomisations (94.0%) over 7 months. There were high levels of engagement with the intervention. Dental visiting data were available for 84.8% of participants (95% CI: 75.3%, 91.1%). Uptake of the optional dental examination within the research assessment battery was low (follow‐up: 12.7%; 95% CI: 7.0%, 21.8%). There were no serious adverse events attributable to the intervention or trial procedures. There were substantially higher rates of dental attendance after nine months in the link work intervention arm, compared to TAU, in both the self‐report (91.7% vs. 26.7%) and NHS BSA (55.3% vs. 12.1%) data. There was also a signal of improved self‐reported oral health‐related quality of life favouring the link work intervention arm.

**Conclusions:**

The trial procedures and link work intervention were found to be feasible, acceptable and safe. The intervention showed promise in terms of clinical outcomes. The effectiveness of the intervention requires evaluation in a larger trial.

**Trial Registration:**

NCT05545228

## 1 Introduction

People with severe mental illness (e.g., psychosis, bipolar disorder) experience profound and multi‐faceted physical health problems [1]. This includes poor oral health with high rates of decayed, missing and filled teeth [2], periodontal disease [3] and oral lesions [4], compared to the general population. This is likely due to elevated risk factors for poor oral health [5] and the iatrogenic effects of psychiatric medications, which can contribute to xerostomia and tooth decay [6]. Poor oral health may place an additional burden and stress on people already living with the challenges of severe mental illness.

Despite their elevated risk of oral health problems, people with severe mental illness are less likely to attend routine dental appointments [7]. This is due to multiple interacting barriers to dental attendance, including demotivation, anxiety and treatment costs [8]. At times, psychiatric symptoms (e.g., paranoia, depression) make dental visiting more challenging [9]. Dental practice operating procedures may disadvantage people with severe mental illness (e.g., short consultations, unforgiving discharge policies) making it harder for them to keep appointments [8]. This may be further exacerbated by a lack of NHS dental services in England, making access to dentistry even more challenging [10]. There is a need to better enable people with severe mental illness to use dental services so that they can receive appropriate treatment.

No evidence‐based, scalable interventions currently exist for improving the oral health of people with severe mental illness [11]. Past clinical evaluations have typically focused on educating people around oral hygiene with no clinically significant effects [12, 13]. One promising area of investigation concerns link work interventions, which aim to enable disempowered groups to navigate the gap between services using support workers, without a professional background. The *Childsmile* initiative in Scotland found that families at risk of poor oral health receiving link work were twice as likely to attend a dental appointment [14]. To the best of the authors' knowledge, there are currently no trials of link work interventions focusing on dental attendance in adults with severe mental illness.

This paper reports on the Mouth Matters in Mental Health Trial, a multi‐site feasibility randomised controlled trial (RCT) exploring the feasibility, acceptability and safety of a link work intervention that aimed to support people with severe mental illness to access a routine dental appointment. Feasibility outcomes included the ability to recruit participants, gather outcome data at follow‐up, and retain participants in the intervention. Secondary outcomes related to oral and mental health outcomes, and dental visiting.

## 2 Methods

### 2.1 Design

A feasibility RCT across three NHS Trusts in the Northwest of England. Individual allocation of participants occurred on a 1:1 basis across two arms: treatment as usual (TAU) or TAU plus the link work intervention. Research assistants completed assessments at baseline and after 9‐months. The researchers accessed routinely collected dental visiting data via the NHS Business Services Authority (BSA), which processes data from NHS dental practices for the purpose of payment. A qualitative process evaluation is reported separately. The trial was pre‐registered (ISRCTN13650779) and has a published protocol [15].

### 2.2 Participants

Eligible participants were in receipt of care from an NHS secondary care mental health service (i.e., Community Mental Health Team, Early Intervention for Psychosis Service) at the point of referral into the trial, but had not attended a routine dental appointment in the past 3 years. This included any planned, non‐urgent dental appointments, resulting in a dental examination, diagnosis, advice or treatment. Emergency dental care (e.g., attendance at A&E) was not included in this definition, although follow‐up routine appointments excluded participation. The choice of 3 years without planned care was based on NICE recall guidance [16], which recommends low risk patients in the UK be seen every years. This ensured that participants were unlikely to be routinely attending a dentist. Eligibility was based on service provision, rather than diagnostic criteria, but the diagnostic composition of the sample was recorded for descriptive purposes. Participants were eligible if aged ≥ 18 and able to provide informed consent. Exclusion criteria included inpatient status, immediate risk to self or others and enrolment on another dental trial. Participants could be referred by mental health staff or self‐refer. All participants provided informed written or audio‐recorded consent.

### 2.3 Randomisation and Blinding

Liverpool Clinical Trials Centre (LCTC) managed the randomisation system which informed the link workers, trial manager and sites leads (JPC, PF, RG) of treatment allocation. Randomisation occurred on a 1:1 ratio, stratified by NHS site, using random allocation blocks. Research assistants, responsible for conducting follow‐up assessments, were masked to treatment allocation. Actions were taken to prevent unblinding throughout the trial (e.g., regular reminders, separate telephone numbers). In cases of unblinding, a separate research assistant was used to ‘re‐blind’ the assessment. Trial statisticians were blind until the statistical analysis plan was finalised.

### 2.4 Treatment

The aim of the link work intervention was to support and empower people with severe mental illness to attend a routine dental appointment. Link workers were support workers situated in the participating mental health NHS trusts, guided by an intervention manual co‐developed through patient and public involvement workshops and underpinned by the COM‐B model. The intervention involved knowledge exchange, demystification, social support and anxiety reduction techniques to strengthen capacity around dental visiting. Link workers helped people to identify dentists, and then book, plan, attend and offer advocacy at dental appointments, and navigate and apply for free/subsidised dental care and financial support, applying motivational interviewing and anxiety reduction techniques.

Link workers could offer up to six sessions with participants over nine months, plus additional brief contacts (e.g., phone calls, texts). Visits were typically 1:1 but could involve supportive others. Appointments were conducted at places of mutual convenience. Link workers were equipped with knowledge of dental health provision and expected to regularly review whether local dentists were currently accepting new patients. Adherence to the manual was monitored using sessional checklists completed by link workers and clinical supervision.

### 2.5 Treated as Usual

TAU was considered as any concomitant support the participant already had to facilitate dental attendance. Given the eligibility criteria, most participants had support from a mental health service. Typically, these services support patients with their mental and physical health, but dental attendance is often not a focus. Participants in TAU were able to access assessment, information and treatment from dental services as normal, but without the support of a designated link worker.

### 2.6 Outcomes

This RCT was primarily concerned with the feasibility, acceptability and safety of the intervention and trial procedures, measured against pre‐specified traffic light criteria. One or more amber or red outcomes would indicate that minor or major adaptations were required to the trial protocol and/or intervention, prior to a full trial. The feasibility criteria included the ability to recruit 84 participants across three sites within seven months (green ≥ 80%. amber 60%–79%. red ≤ 59%); the percentage of participants with available self‐report or BSA dental visiting data (green ≥ 90%, amber 60%–89% and red ≤ 59%); the percentage of participants completing a dental examination (green ≥ 80%, amber 60%–79% and red: ≤ 59%); and the percentage of participants receiving ≥ 1 link work sessions during a nine‐month intervention window (green ≥ 80%, amber 60%–79% and red ≤ 59%). Safety was assessed through the monitoring of research‐related serious adverse events (SAEs), reviewed by an independent Trial Steering Committee (TSC).

### 2.7 Assessments

Research assistants met participants face‐to‐face or remotely (e.g., telephone, online) to complete clinical assessments, at baseline and after nine‐months. The pre‐specified proposed primary outcome for a definitive trial was attendance at a planned dental appointment, using the item ‘Have you attended a dental service since the baseline assessment (9 months ago)? This would include a routine appointment with a dentist or special care dentistry service. It could also include a planned appointment at a dental hospital. However, it would not include an emergency dental appointment’. Researchers asked participants to confirm the nature and timing of appointments.

Planned dental appointments were also assessed through records held by the NHS BSA. NHS England collects information on NHS dental visits and treatment through the NHS BSA, which is used to remunerate dental practices for work completed. Participants provided informed consent for the research team to access their NHS BSA data, which was identified using their surname, first initial, date of birth and gender. The NHS BSA data included whether a care appointment had taken place, the nature of the treatment(s), and NHS payments to the dentist for treatment. This data were extracted 2 months after the final treatment window had closed to allow time for dentists to submit payment claims to the NHS BSA. In the UK, there are both private and NHS dentists. The NHS BSA data could only identify NHS, and not private dental practice visits.

Other secondary outcomes included the Oral Health Impact Profile, 14‐item version [17]; the Brief Pain Inventory, Short Form [18]; the Manchester Orofacial Pain Disability Scale [19]; the Modified Dental Anxiety Scale [20]; the Patient Health Questionnaire [21]; the Rosenberg Self‐Esteem Scale [22]; and the EuroQol 5 Dimension (EQ‐5D‐5L [23]). Self‐efficacy around dental visiting was assessed using the item ‘How confident are you that you will be able to attend a dental appointment?’ (adapted from Ref. [24]). The assessment also measured oral self‐hygiene behaviours and risk factors for poor oral health (e.g., alcohol, cigarette use). The MINI diagnostic interview [25] assessed the diagnostic composition of participants. Index of Multiple Deprivation (IMD) acted as a metric of socioeconomic deprivation.

Participants were offered the opportunity to complete an optional dental examination as part of the research assessment battery. If participants consented, a dental therapist accompanied the research assistant to the baseline and nine‐month assessments and used portable equipment to examine the oral cavity, including the number of decayed, missing and filled teeth (DMFT); pulpal involvement, ulceration due to trauma, fistula and abscess (PUFA); and levels of plaque (modified plaque score). The aim was to explore the feasibility of assessing these oral health outcomes. The examination was made optional so as not to prohibit participation from people with dental anxiety. Uptake of the dental examination was measured as a feasibility question. Dentists trained, calibrated and supervised the dental therapist.

### 2.8 Statistical Analysis

All outcomes reported were prespecified in the Statistical Analysis Plan (SAP), prepared by the trial statisticians (EG, GB), following LCTC standard operating procedures and prior to accessing allocations. The sample size allowed for ≥ 70% of the expected participants to be recruited, with the lower end of the 95% confidence interval above the 60% amber/red cut‐off point. Descriptive data are presented as mean and standard deviations for continuous variables, and frequencies and percentages for categorical variables at baseline and follow‐up, overall and by treatment arm. Analyses were performed according to the intention‐to‐treat approach, including all participants randomised, regardless of adherence to the study protocol.

Feasibility outcomes are presented descriptively and compared to the traffic light criteria as defined in the protocol. The efficacy of intervention was not investigated. However, descriptive summaries are presented overall and split by treatment arm for all proposed outcome measures, to inform the design of the definitive trial. Data have been reported according to the updated CONSORT2010 statement for randomised pilot and feasibility trials. Statistical analyses were conducted in SAS (v9.4).

## 3 Results

**FIGURE 1:**
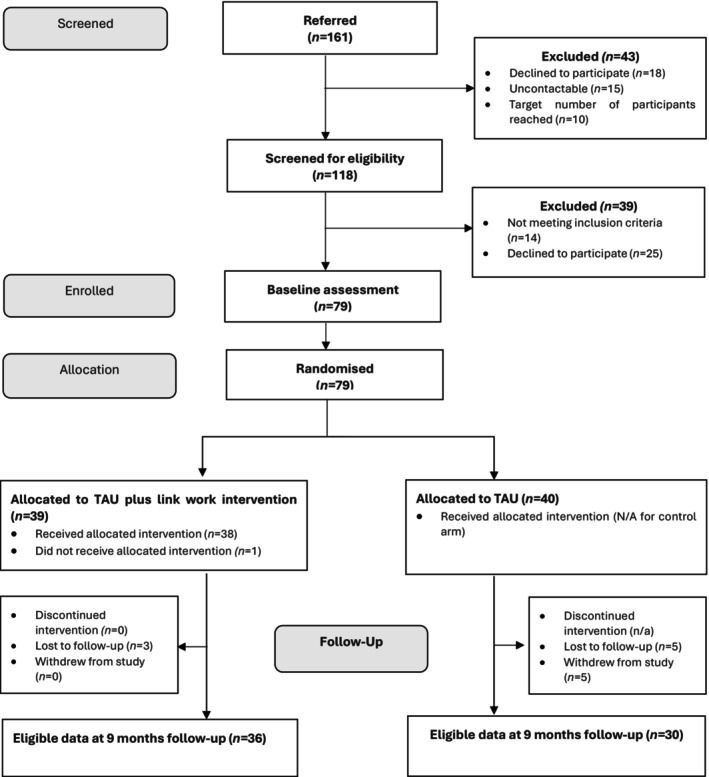
CONSORT diagram.

One hundred and sixty‐one people were referred into the trial between September 2022 and April 2023 (first randomisation: 04 November 2022), with follow‐up assessments concluding in February 2024. One hundred and eighteen people were screened for eligibility, resulting in 79 randomisations (40 in TAU; 39 in TAU plus link work). Figure 1 shows the CONSORT diagram. Five participants withdrew from the study after the baseline assessment (all in the TAU arm). Sixty‐six participants completed the follow‐up self‐report assessments after nine months (30 in TAU; 36 in TAU plus the link work intervention). Research assistants were unblinded on 11 occasions, but with re‐masking/drop out, only five follow‐ups were unblind at assessment.

### 3.1 Sample Information

There was minimal difference between the arms of the trial in terms of baseline demographics and concomitant treatments (Table S1). Over 61% of participants lived in the two most socially deprived areas in the UK according to the IMD. There were high rates of self‐reported risk factors for poor oral health, including smoking, alcohol use, drug use, teeth grinding and dry mouth. At baseline, most participants (89.9%) were taking psychotropic medications and a third were engaging in psychological therapies (34.2%). Fifty‐one participants (64.6%) reported a past psychiatric hospital admission. Three were admitted to an inpatient ward during the trial. Table S2 shows the diagnostic composition of the sample based on the MINI. A high proportion of participants met the criteria for an anxiety, psychotic disorder or substance abuse disorder.

### 3.2 Feasibility Outcomes

The three sites recruited 79 out of the target 84 participants (94.0%; green). Only one site failed to recruit to target but achieved 23 out of 28 participants (82.1%). Dental visiting data (self‐report or BSA) at follow‐up was available in 67 of 79 participants (84.8%; 95% CI: 75.3%, 91.1%), which was slightly under the feasibility target of 90% (amber). Uptake of the dental examination was generally low at baseline (*n* = 25/79; 31.7%; 95% CI 22.5%, 42.6%) and reduced further at follow‐up (*n* = 10/79; 12.7%; 95% CI 7.0%, 21.8%), attesting to a lack of feasibility in this area (red). Across both time points, common reasons for not completing the dental examination included participants not consenting (*n =* 24), being unable to meet with the dental therapist (*n =* 22) or opting for remote assessment (*n* = 8). In the treatment arm, 38 out of 39 participants (97.4%; 95% CI 86.8%, 99.6%) completed ≥ 1 session of the link work intervention (green).

### 3.3 Safety Data

The trial identified 56 AEs across 29 participants (Table S3). There were 37 AEs across 17 participants in the link work arm and 19 AEs across 12 participants in the TAU arm, potentially due to increased monitoring during the link work intervention. Sixteen SAEs were identified across eight participants during the trial, with eight SAEs in four participants in each arm (Table S4). All SAEs were deemed to be unrelated to the study procedures and intervention by the independent TSC.

### 3.4 Intervention Information

In participants receiving the link work intervention, the average number of sessions attended was 4.1 (SD: 2.0). Seventeen participants (43.6%) attended all six sessions. The average length of sessions was 75.2 min (SD: 44.5), but they varied considerably in length (15–245 min). One site had a higher average length of sessions (mean: 102 min, SD: 54.9) potentially due to its large geographical footprint. Table S5 shows the focus of sessions.

### 3.5 Dental Attendance

Thirty‐three out of 36 participants (91.7%) in the link work arm self‐reported that they had attended a planned dental appointment, compared to eight out of 30 participants (26.7%) in the TAU arm. Rates of dental attendance were lower in the NHS BSA data, but there was still a large difference between the two arms of the trial: 21 out of 38 participants (55.3%) in the link work arm were identified as having attended a dentist, compared to 4 out of 33 participants (12.1%) in the TAU arm.

Twenty‐eight participants self‐reported attending an NHS dentist and also had a data request submitted to the NHS BSA. In 17 (60.7%) cases, there was agreement between the data sources; the NHS BSA also indicated that the person had seen an NHS dentist. Of the eight participants who self‐reported attending a private dentist, the NHS BSA data suggested that two (25.0%) were misattributed NHS dental appointments. Two participants self‐reported being unsure of whether they had attended a private or NHS dental appointment, and the NHS BSA data indicated that both (100%) were NHS dental appointments. Therefore, there was some discrepancy between the data sources.

Participants in the link work arm who had successfully been to a dentist self‐reported attending an average of 3.2 planned dental appointments (SD: 1.9, *n*: 33), compared to 2.6 appointments in those who had attended a dentist in the TAU arm (SD 1.5, *n*: 8). Twenty‐four out of 35 (68.6%) participants receiving the link work intervention successfully accessed free or subsidised dental care, compared with only three out of 30 (10.0%) participants in the TAU arm.

### 3.6 Other Secondary Outcomes

Clinical outcomes are summarised in Table 1. There was an improvement in oral health quality of life in both arms, which was considerably larger in participants receiving the link work intervention (6.7 vs. 1.9 points). The data showed little change in dental anxiety, confidence in dental visiting, and depression scores at follow‐up, slightly favouring the link work intervention arm. Conversely, there was a slight improvement in self‐esteem in participants receiving only TAU. General quality of life as measured by EQ‐5D‐5L was relatively stable in both arms. Too few participants completed the dental examination to provide meaningful interpretation of the results.

**TABLE 1:** Self‐report assessments and dental examination scores at baseline and follow‐up.

Assessment	TAU	TAU + link work	Overall
**OHIP‐14**			
Baseline, mean (SD, *n*)	23.7 (15.1, 40)	24.7 (15.1, 39)	24.2 (15.0, 79)
Follow‐up, mean (SD, *n*)	19.8 (12.6, 27)	15.6 (13.0, 34)	17.5 (12.9, 61)
Change in scores, mean (SD, *n*)	−1.9 (8.6, 27)	−6.7 (12.5, 34)	−4.6 (11.1, 61)
**Dental visiting confidence**			
Baseline, mean (SD, *n*)	5.5 (1.8, 40)	5.2 (1.9, 39)	5.3 (1.9, 79)
Follow‐up, mean (SD, *n*)	4.9 (2.2, 27)	5.2 (1.6, 34)	5.1 (1.9, 61)
Change in scores, mean (SD, *n*)	−0.3 (2.4, 27)	0.3 (2.1, 34)	0.0 (2.2, 61)
**MDAS**			
Baseline, mean (SD, *n*)	16.1 (5.8, 39)	14.3 (5.6, 39)	15.2 (5.8, 78)
Follow‐up, mean (SD, *n*)	17.2 (6, 27)	14.1 (4.7, 33)	15.5 (5.5, 60)
Change in scores, mean (SD, *n*)	0.4 (4.0, 26)	−0.1 (5.8, 33)	0.1 (5.1, 59)
**PHQ‐9**			
Baseline, mean (SD, *n*)	14.2 (7.1, 39)	13.7 (7.7, 38)	13.9 (7.4, 77)
Follow‐up, mean (SD, *n*)	12.7 (7.4, 26)	12.9 (7.6, 32)	12.8 (7.4, 58)
Change in scores, mean (SD, *n*)	0.0 (6.0, 26)	−0.4 (4.8, 32)	−0.2 (5.4, 58)
**RSES**			
Baseline, mean (SD, *n*)	11.3 (6.0, 40)	13.6 (5.0, 39)	12.4 (5.6, 79)
Follow‐up, mean (SD, *n*)	13.8 (6.0, 27)	14.6 (5.3, 33)	14.3 (5.6, 60)
Change in scores, mean (SD, *n*)	2.3 (6.7, 27)	0.6 (4.6, 33)	1.4 (5.7, 60)
**EQ‐5D‐5L—Index Score**			
Baseline, mean (SD, *n*)	0.5 (0.3, 40)	0.5 (0.3, 39)	0.5 (0.3, 79)
Follow‐up, mean (SD, *n*)	0.5 (0.3, 27)	0.7 (0.3, 32)	0.6 (0.3, 59)
Change in scores, mean (SD, *n*)	0.0 (0.3, 27)	0.1 (0.3, 32)	0.1 (0.3, 59)
**EQ‐5D‐5L—Visual Analogue Scale**			
Baseline, mean (SD, *n*)	53.0 (24.2, 40)	53.9 (23.2, 39)	53.4 (23.6, 79)
Follow‐up, mean (SD, *n*)	49.4 (15.8, 27)	54.8 (24.1, 32)	52.3 (20.7, 59)
Change in scores, mean (SD, *n*)	−0.7 (24.6, 27)	0.5 (22.2, 32)	−0.1 (23.1, 59)
**Dental exam: DMFT Score**			
Baseline, mean (SD, *n*)	17.6 (9.7, 14)	13.9 (8.3, 11)	16.0 (9.1, 25)
Follow‐up, mean (SD, *n*)	15.3 (12.8, 4)	13.2 (7.9, 6)	14.0 (9.5, 10)
Change in scores, mean (SD, *n*)	−2.8 (2.2, 4)	−0.7 (2.9, 6)	−1.5 (2.8, 10)
**Dental exam: Plaque score**			
Baseline, mean (SD, *n*)	18.1 (9.0, 14)	17.5 (6.3, 11)	17.9 (7.8, 25)
Follow‐up, mean (SD, *n*)	9.5 (9.0, 4)	10.2 (5.8, 6)	9.9 (6.7, 10)
Change in scores, mean (SD, *n*)	0.8 (4.3, 4)	−7.0 (5.2, 6)	−3.9 (6.1, 10)
**Dental exam: PUFA score**			
Baseline, mean (SD, *n*)	5.4 (7.6, 14)	1.5 (1.6, 11)	3.6 (6.0, 25)
Follow‐up, mean (SD, *n*)	4.7 (6.4, 3)	1.0 (1.3, 6)	2.2 (3.8, 9)
Change in scores, mean (SD, *n*)	−0.3 (2.5, 3)	−1.2 (1.2, 6)	−0.9 (1.6, 9)

*Note:* BPI, Brief Pain Inventory; DMFT, decayed, missing and filled teeth; EQ‐5D‐5L, EuroQol 5 Dimension; MDAS, Modified Dental Anxiety Scale; OHIP‐14, Health Impact Profile; PHQ‐9, Patient Health Questionnaire; PUFA, pulpal involvement, ulceration due to trauma, fistula and abscess; RSES, Rosenberg Self‐Esteem Scale.

Additional dental outcomes are presented in Table S6. The number of participants reporting orofacial pain reduced between baseline and follow‐up, particularly in the link work intervention arm, but numbers were small. No major changes were observed in the frequency or length of tooth brushing, inter‐dental brushing or use of fluoridated mouthwash. Five participants in each arm attended an emergency dental appointment.

## 4 Discussion

This is the first feasibility RCT of a link work intervention to facilitate dental attendance in people with severe mental illness. The trial demonstrated feasibility in terms of recruitment rates, engagement with the intervention and the ability to collect dental attendance data. However, uptake of an optional dental examination was low, indicating a lack of feasibility in this area. There were no safety concerns: rates of SAEs were to be expected given the recruitment of people with severe mental illness, and none were attributed to the trial procedures or intervention. Overall, the findings suggest that, with some adjustments, a full trial of a link work intervention to enable dental access in people with severe mental illness would be feasible.

As the primary focus was on feasibility, the trial was not powered to determine effectiveness. Nonetheless, preliminary signals in outcomes were investigated. 91.7% of participants in the link work intervention arm self‐reported that they had attended a planned dental appointment after nine months, compared to 26.7% in the TAU arm. Rates of dental attendance were lower in the NHS BSA data but still demonstrated higher rates of dental attendance in the link work intervention arm (55.3% vs. 12.1%). The results should be interpreted within the context of the limited availability of dental services in England generally [10]. There was greater improvement in oral health‐related quality of life in the link work arm compared to TAU (6.7 vs. 1.9 points), tentatively suggesting positive clinical effects of dental attendance. A three‐to‐five‐point change on the OHIP‐14 is thought to be clinically meaningful [26]. Together with the low rates of missing data, oral health quality of life is one potential candidate for a primary outcome in a full trial.

Past approaches to increase regular health service usage among vulnerable populations have led to the unintended consequence of increasing inequalities for disadvantaged groups. A systematic review showed that walk‐in centres and telephone advice services advantage healthy middle‐class patients [27]. Additionally, there is some evidence that link work interventions in primary care have failed to target those geographical areas with the greatest need [28]. This does not appear to be true of the current trial, where the majority of participants lived in the most socially deprived areas of the UK and the level of oral health need and complexity were high.

The findings contribute to the existing literature [2, 3] highlighting that oral health inequalities exist among people with severe mental illness. At baseline, 38.0% of the sample had experienced orofacial pain for at least 24 h in the past month. Over 25% had been to an emergency dental appointment in the past 3 years. The sample also experienced multiple risk factors for poor oral health and low rates of oral hygiene behaviours.

There are several possible explanations for the discrepancy in the self‐report and NHS BSA dental visiting data. First, it is possible that some dental surgeries had not yet submitted their data to the NHS BSA, resulting in missing entries. Second, there may have been some discrepancy in the personal information used to extract NHS BSA data. For example, if a participant changed their name, this may have led to missing data. Third, only the self‐report and not the NHS BSA data was able to identify private dental appointments. Fourth, there may have been some inaccuracies in the self‐report data due to confusion around the definition of a routine dental appointment. Consequently, it is likely that both data types have their own strengths and limitations, and it will be important to retain both in a definitive trial.

Strengths of this feasibility RCT are that it was conducted across three sites and had dental clinical record linkage, alongside self‐report data and a pre‐registered protocol. There was low engagement with an optional dental examination, which has implications for future research. Alternative approaches (e.g., self‐counting of teeth) may be more acceptable in this population and easier to administer, but require investigation and evaluation. Inclusion of the EQ‐5D‐5L in the assessment battery may help to facilitate a future economic evaluation.

## 5 Conclusion

This feasibility RCT supports the feasibility, acceptability and safety of a link work intervention to help people attend routine dental appointments to facilitate appropriate care. The intervention shows promise with signals around improved clinical outcomes. The next step is to explore the effectiveness and cost‐effectiveness of the link work intervention within a full trial.

## Author Contributions

All authors contributed to the development and refinement of the project protocol. Jasper Palmier‐Claus and Rebecca Harris led the trial. Jasper Palmier‐Claus, Girvan Burnside, Vishal R. Aggarwal, Rebecca Harris, Sarah Procter, Robert Griffiths, Paul French, Louise Laverty, Fiona Lobban, Katherine Berry, Christopher Lodge and David Shiers are all grant holders. Claire Hilton and Abigail Morris were trial managers. Efstathia Gkioni was the trial statistician, drafted the statistical analysis plan, and carried out the final analysis, supervised by Girvan Burnside. Rebecca Golby, Fanni Fazekas, Kyriakos Valemis, Alison Dawber, Antonia Perry and Farah Lunat were responsible for data collection at the sites. Fiona Lobban and Louise Laverty led the qualitative evaluation. Connie Newens, Pauline Mupinga and Eirian Kerry delivered the link work intervention. Vishal R. Aggarwal and Sarah Procter offered training and supervision around the dental examination. Emma Elliott supported the manual and dental examination design. Christopher Lodge and David Shiers offered lived and carer experiences to the project team.

## Ethics Statement

This trial has received approval from an NHS research ethics committee (Wales Research Ethics Committee 2; ID: 304696). The authors assert that all procedures contributing to this work comply with the ethical standards of the relevant national and institutional committees on human experimentation and with the Helsinki Declaration of 1975, as revised in 2013.

## Consent

Study consent forms and participant information sheets are available on request.

## Conflicts of Interest

D.S. is expert advisor to the NICE centre for guidelines; the views expressed are the authors' and not those of NICE.

## Supporting information


**Table S1.** Results of the MINI diagnostic interview at baseline.
**Table S2.** Summary of adverse events (AE) by category.
**Table S3.** Summary of serious adverse events (SAE) by category.
**Table S4.** Primary focus of link work sessions.
**Table S5.** Dental outcomes at baseline and follow‐up.

## Data Availability

An anonymised final trial dataset will be available following publication of the study, at the discretion of the authors.
