# Supplementary material for: A Link Work Intervention to Facilitate Dental Visiting in People With Severe Mental Illness: A Two‐Arm, Multi‐Site, Assessor Blind, Randomised Feasibility Trial With Dental Record Linkage
Source: Community Dent Oral Epidemiol. 2025 Aug 4;53(5):580–6. doi: 10.1111/cdoe.70002 (PMC12423352; doi:10.1111/cdoe.70002)
Supplement: Supplementary file 1 — Table S1. Results of the MINI diagnostic interview at baseline. Table S2. Summary of adverse events (AE) by category. Table S3. Summary of serious adverse events (SAE) by category. Table S4. Primary focus of link work sessions. Table S5. Dental outcomes at baseline and follow‐up. [file CDOE-53-580-s001.docx]

| Supplementary Table 1. Sample characteristics at baseline. | |  |  |  |
| --- | --- | --- | --- | --- |
| **Demographic** | **Summary** | **TAU (*n=*40),** *n*(%) | **TAU plus link work intervention (*n*=39)**, *n*(%) | **Overall (*n*=79)**, *n*(%) |
| Gender | Female | 21 (52.5) | 15 (38.5) | 36 (45.6) |
|  | Male | 18 (45.0) | 23 (59.0) | 41 (51.9) |
|  | Other | 1 (2.5) | 1 (2.6) | 2 (2.5) |
| Age, years | Mean (S.D.) | 39.2 (13.9) | 41.8 (13.5) | 40.5 (13.6) |
|  | Min-Max | 18.0 to 65.0 | 22.0 to 66.0 | 18.0 to 66.0 |
| Sexuality | Heterosexual | 36 (90.0) | 38 (97.4) | 74 (93.7) |
|  | Gay / Lesbian | 2 (5.0) | 0 (0.0) | 2 (2.5) |
|  | Other | 2 (5.0) | 1 (2.6) | 3 (3.8) |
| Marital status | Single | 28 (70.0) | 26 (66.7) | 54 (68.4) |
|  | Partnered | 10 (25.0) | 13 (33.3) | 23 (29.1) |
|  | Other | 2 (5.0) | 0 (0.0) | 2 (2.5) |
| Ethnicity | White British | 31 (79.5) | 28 (71.8) | 59 (75.6) |
|  | Black British | 3 (7.7) | 1 (2.6) | 4 (5.1) |
|  | White other | 3 (7.7) | 1 (2.6) | 4 (5.1) |
|  | Asian / Asian British | 0 (0.0) | 4 (10.3) | 4 (5.1) |
|  | Irish Gypsy or Traveller | 1 (2.6) | 1 (2.6) | 2 (2.6) |
|  | Other ethnicity | 1 (2.6) | 4 (10.3) | 5 (6.4) |
|  | Missing | 1 | 0 | 1 |
| Highest level of education | No education | 6 (15.0) | 10 (26.3) | 16 (20.5) |
|  | GCSEs or similar | 17 (42.5) | 16 (42.1) | 33 (42.3) |
|  | A level or similar | 13 (32.5) | 9 (23.7) | 22 (28.2) |
|  | Degree or higher | 4 (10.0) | 3 (7.9) | 7 (9.0) |
|  | Missing | 0 | 1 | 1 |
| Paid employment | No | 36 (90.0) | 32 (82.1) | 68 (86.1) |
|  | Yes | 4 (10.0) | 7 (17.9) | 11 (13.9) |
| Receipt of benefits | No | 4 (10.0) | 3 (7.7) | 7 (8.9) |
|  | Yes | 36 (90.0) | 36 (92.3) | 72 (91.1) |
| Social deprivation IMD rank: decile | 1: 10% most deprived | 13 (32.5) | 16 (42.1) | 29 (37.2) |
|  | 2: 10% to 20% | 10 (25.0) | 9 (23.7) | 19 (24.4) |
|  | 3: 20% to 30% | 6 (15.0) | 5 (13.2) | 11 (14.1) |
|  | 4: 30% to 40% | 4 (10.0) | 2 (5.3) | 6 (7.7) |
|  | 5: 40% to 50% | 2 (5.0) | 2 (5.3) | 4 (5.1) |
|  | 6: 50% to 60% | 0 (0.0) | 1 (2.6) | 1 (1.3) |
|  | 7: 60% to 70% | 0 (0.0) | 1 (2.6) | 1 (1.3) |
|  | 8: 70% to 80% | 4 (10.0) | 2 (5.3) | 6 (7.7) |
|  | 9: 80% to 90% | 1 (2.5) | 0 (0.0) | 1 (1.3) |
|  | 10: 10% least deprived | 0 (0.0) | 0 (0.0) | 0 (0.0) |
|  | Missing | 0 | 1 | 1 |
| Approximate age when first came into contact with mental health services, years | Mean (*S.D*.) | 27.5 (13.2) | 28.7 (12.7) | 28.1 (12.9) |
|  | Min-Max | 10.0 to 58.0 | 9.0 to 58.0 | 9.0 to 58.0 |
|  | Missing, *n* | 1 | 0 | 1 |
| Referring clinical service | Early Intervention Team | 20 (50) | 18 (46.2) | 38 (48.1) |
|  | Community Mental Health Team | 20 (50) | 21 (53.8) | 41 (51.9) |
| Smoking/chewing tobacco products | Yes: daily | 18 (45.0) | 24 (61.5) | 42 (53.2) |
|  | Yes: occasionally but not every day | 3 (7.5) | 2 (5.1) | 5 (6.3) |
|  | No: but I have in the past | 13 (32.5) | 7 (17.9) | 20 (25.3) |
|  | No: I have never used tobacco | 6 (15.0) | 6 (15.4) | 12 (15.2) |
| Drinking alcohol | No | 16 (40.0) | 18 (46.2) | 34 (43.0) |
|  | Yes | 24 (60.0) | 21 (53.8) | 45 (57.0) |
| Taking recreational drugs | No | 30 (75.0) | 28 (71.8) | 58 (73.4) |
|  | Yes | 10 (25.0) | 11 (28.2) | 21 (26.6) |
| Grinding teeth during the day or at night | No | 28 (70.0) | 23 (59.0) | 51 (64.6) |
|  | Yes | 12 (30.0) | 16 (41.0) | 28 (35.4) |
| Mouth usually feeling dry | No | 11 (27.5) | 18 (46.2) | 29 (36.7) |
|  | Yes | 29 (72.5) | 21 (53.8) | 50 (63.3) |
| Taking psychiatric medications | No | 6 (15.0) | 2 (5.1) | 8 (10.1) |
|  | Yes | 34 (85.0) | 37 (94.9) | 71 (89.9) |
| Receiving psychological or talking therapies | No | 28 (70.0) | 24 (61.5) | 52 (65.8) |
|  | Yes | 12 (30.0) | 15 (38.5) | 27 (34.2) |
| ≥1 past psychiatric admission(s) | No | 18 (45.0) | 10 (25.6) | 28 (35.4) |
|  | Yes | 22 (55.0) | 29 (74.4) | 51 (64.6) |

| Supplementary Table 2. Results of the MINI diagnostic interview at baseline. | | | |  |  |
| --- | --- | --- | --- | --- | --- |
| **Question** | **Summary** | **TAU**,  *n* (%) | **TAU plus link work intervention**,  *n* (%) | **Overall*,*** *n* (%) |  |
|  |  |  |  |  |  |
|  |  |  |  |  |  |
| Major depressive disorder (current) | No | 32 (86.5) | 32 (86.5) | 64 (86.5) |  |
|  | Yes | 5 (13.5) | 5 (13.5) | 10 (13.5) |  |
|  | N (*n* missing) | 37 (3) | 37 (2) | 74 (5) |  |
| Major depressive disorder (past) | No | 26 (70.3) | 30 (81.1) | 56 (75.7) |  |
|  | Yes | 11 (29.7) | 7 (18.9) | 18 (24.3) |  |
|  | N (*n* missing) | 37 (3) | 37 (2) | 74 (5) |  |
| Major depressive disorder (recurrent) | No | 32 (86.5) | 32 (88.9) | 64 (87.7) |  |
|  | Yes | 5 (13.5) | 4 (11.1) | 9 (12.3) |  |
|  | N (*n* missing) | 37 (3) | 36 (3) | 73 (6) |  |
| Bipolar I disorder (current) | No | 36 (97.3) | 36 (97.3) | 72 (97.3) |  |
|  | Yes | 1 (2.7) | 1 (2.7) | 2 (2.7) |  |
|  | N (*n* missing) | 37 (3) | 37 (2) | 74 (5) |  |
| Bipolar I disorder (past) | No | 28 (75.7) | 28 (75.7) | 56 (75.7) |  |
|  | Yes | 9 (24.3) | 9 (24.3) | 18 (24.3) |  |
|  | N (*n* missing) | 37 (3) | 37 (2) | 74 (5) |  |
| Panic disorder (current) | No | 27 (71.1) | 22 (59.5) | 49 (65.3) |  |
|  | Yes | 11 (28.9) | 15 (40.5) | 26 (34.7) |  |
|  | N (*n* missing) | 38 (2) | 37 (2) | 75 (4) |  |
| Panic disorder (lifetime) | No | 20 (54.1) | 18 (48.6) | 38 (51.4) |  |
|  | Yes | 17 (45.9%) | 19 (51.4) | 36 (48.6) |  |
|  | N (*n* missing) | 37 (3) | 37 (2) | 74 (5) |  |
| Agoraphobia (current) | No | 14 (36.8) | 23 (62.2) | 37 (49.3) |  |
|  | Yes | 24 (63.2) | 14 (37.8) | 38 (50.7) |  |
|  | N (*n* missing) | 38 (2) | 37 (2) | 75 (4) |  |
| Social anxiety - (current) | No | 16 (42.1) | 22 (59.5) | 38 (50.7) |  |
|  | Yes | 22 (57.9) | 15 (40.5) | 37 (49.3) |  |
|  | N (*n* missing) | 38 (2) | 37 (2) | 75 (4) |  |
| Obsessive Compulsive Disorder (current) | No | 23 (60.5) | 27 (73.0) | 50 (66.7) |  |
|  | Yes | 15 (39.5) | 10 (27.0) | 25 (33.3) |  |
|  | N (*n* missing) | 38 (2) | 37 (2) | 75 (4) |  |
| Post Trauma Stress Disorder | No | 23 (60.5) | 27 (75.0) | 50 (67.6) |  |
|  | Yes | 15 (39.5) | 9 (25.0) | 24 (32.4) |  |
|  | N (*n* missing) | 38 (2) | 36 (3) | 74 (5) |  |
| Alcohol use disorder (past 12 months) | No | 25 (67.6) | 29 (78.4) | 54 (73.0) |  |
|  | Yes | 12 (32.4) | 8 (21.6) | 20 (27.0) |  |
|  | N (*n* missing) | 37 (3) | 37 (2) | 74 (5) |  |
| Substance abuse disorder (past 12 months | No | 24 (64.9) | 23 (65.7) | 47 (65.3) |  |
|  | Yes | 13 (35.1) | 12 (34.3) | 25 (34.7) |  |
|  | N (*n* missing) | 37 (3) | 35 (4) | 72 (7) |  |
| Any psychotic disorder (current) | No | 30 (81.1) | 25 (69.4) | 55 (75.3) |  |
|  | Yes | 7 (18.9) | 11 (30.6) | 18 (24.7) |  |
|  | N (*n* missing) | 37 (3) | 36 (3) | 73 (6) |  |
| Any psychotic disorder (lifetime) | No | 19 (51.4) | 15 (41.7) | 34 (46.6) |  |
|  | Yes | 18 (48.6) | 21 (58.3) | 39 (53.4) |  |
|  | N (*n* missing) | 37 (3) | 36 (3) | 73 (6) |  |
| Generalised anxiety disorder (current) | No | 16 (44.4) | 16 (44.4) | 32 (44.4) |  |
|  | Yes | 20 (55.6) | 20 (55.6) | 40 (55.6) |  |
|  | N (*n* missing) | 36 (4) | 36 (3) | 72 (7) |  |

| Supplementary Table 3. Summary of adverse events (AE) by category. | | |  |  |  |  |
| --- | --- | --- | --- | --- | --- | --- |
| Adverse event term | TAU | | TAU plus link work intervention | | Total | |
|  | Events | Participants | Events | Participants | Events | Participants |
|  | *n* | *n* (%) | *n* | *n* (%) | *n* | *n* (%) |
| Mental health | 4 | 3 (7.5) | 6 | 4 (10.3) | 10 | 7 (8.9) |
| Self-harm | 2 | 1 (2.5) | 7 | 6 (15.4) | 9 | 7 (8.9) |
| Unintended injury | 1 | 1 (2.5) | 1 | 1 (2.6) | 2 | 2 (2.5) |
| Missing person | 0 | 0 (0.0) | 1 | 1 (2.6) | 1 | 1 (1.3) |
| Oral health | 3 | 2 (5.0) | 4 | 4 (10.3) | 7 | 6 (7.6) |
| Physical altercation | 1 | 1 (2.5) | 1 | 1 (2.6) | 2 | 2 (2.5) |
| Other physical health | 8 | 7 (17.5) | 17 | 9 (23.1) | 25 | 16 (20.3) |
| Total | 19 | 12 (30.0) | 37 | 17 (43.6) | 56 | 29 (36.7) |
|  |  |  |  |  |  |  |
| Note: AEs include serious adverse events. | | |  |  |  |  |

| Supplementary Table 4. Summary of serious adverse events (SAE) by category. | | |  |  |  |  |
| --- | --- | --- | --- | --- | --- | --- |
| SAE category | TAU | | TAU plus link work intervention | | Total | |
|  | Events | Participants | Events | Participants | Events | Participants |
|  | *n* | *n* (%) | *n* | *n* (%) | *n* | *n* (%) |
| Mental health | 3 | 2 (5.0) | 4 | 3 (7.7) | 7 | 5 (6.3) |
| Self-harm | 2 | 1 (2.5) | 1 | 1 (2.6) | 3 | 2 (2.5) |
| Unintended injury | 0 | 0 (0.0) | 0 | 0 (0.0) | 0 | 0 (0.0) |
| Missing person | 0 | 0 (0.0) | 0 | 0 (0.0) | 0 | 0 (0.0) |
| Oral health | 0 | 0 (0.0) | 0 | 0 (0.0) | 0 | 0 (0.0) |
| Physical altercation | 1 | 1 (2.5) | 0 | 0 (0.0) | 1 | 1 (1.3) |
| Other physical health | 2 | 2 (5.0) | 3 | 2 (5.1) | 5 | 4 (5.1) |
| Total | 8 | 4 (10.0) | 8 | 4 (10.3) | 16 | 8 (10.1) |
|  |  |  |  |  |  |  |
| Note: All SAEs were unrelated to the study protocols and intervention. | | |  |  |  |  |

| Supplementary Table 5. Primary focus of link work sessions. |  |  |  |
| --- | --- | --- | --- |
| **Component of intervention** | **Sessions where component was the primary focus, *n*** | **Participants receiving component of the intervention, *n* (%)** |  |
|  |  |  |  |
| Engagement | 34 | 24 (63.2) |  |
| Risk assessment/management/crisis responding | 4 | 3 (7.9) |  |
| Assessment of oral health / visiting behaviour | 17 | 17 (44.7) |  |
| Support around cost of dental care | 11 | 8 (21.1) |  |
| Support around finding a dentist | 21 | 19 (50.0) |  |
| Support around barriers to attending | 20 | 13 (34.2) |  |
| Visiting a dentist with client | 92 | 31 (81.6) |  |
| Information sharing on oral health relevant to a dental visit | 4 | 3 (7.9) |  |
| Understanding and/or coping with anxiety | 32 | 18 (47.4) |  |
| Information about what to expect during a dental visit | 31 | 21 (55.3) |  |
| Using readiness to change rulers | 2 | 1 (2.6) |  |
| Exploring dental visiting in the context of life goals | 10 | 9 (23.7) |  |
| Reviewing the advantages and disadvantages of dental visiting | 2 | 2 (5.3) |  |
| Reviewing/debriefing of dental visit(s) | 48 | 23 (60.5) |  |
| Planning for the future | 46 | 27 (71.1) |  |
| Other | 21 | 15 (39.5) |  |
|  |  |  |  |
| *Note: Link workers were asked to record up to three primary focuses for each session of the link work intervention. The maximum number of link work sessions per participant was six.* | | |  |

| Supplementary Table 6. Dental outcomes at baseline and follow-up. | | | | | | | |
| --- | --- | --- | --- | --- | --- | --- | --- |
|  |  | Baseline | | | Follow-up | | |
| Variable |  | TAU, *n* (%) | TAU + Link work, *n* (%) | Overall, *n* (%) | TAU, *n* (%) | TAU + Link work, *n* (%) | Overall, *n* (%) |
| Any pain on day of assessment* | No | 30 (75.0) | 26 (68.4) | 56 (71.8) | 14 (51.9) | 26 (78.8) | 40 (66.7) |
|  | Yes | 10 (25.0) | 12 (31.6) | 22 (28.2) | 13 (48.1) | 7 (21.2) | 20 (33.3) |
|  | N (*n* missing) | 40 (0) | 38 (1) | 78 (1) | 27 (13) | 33 (6) | 60 (19) |
| Any orofacial pain on day of assessment* | No | 34 (85.0) | 30 (78.9) | 64 (82.1) | 21 (77.8) | 29 (87.9) | 50 (83.3) |
|  | Yes | 6 (15.0) | 8 (21.1) | 14 (17.9) | 6 (22.2) | 4 (12.1) | 10 (16.7) |
|  | N (*n* missing) | 40 (0) | 38 (1) | 78 (1) | 27 (13) | 33 (6) | 60 (19) |
| Pain in face, mouth or jaws for >24 hours in past month** | No | 25 (62.5) | 24 (61.5) | 49 (62.0) | 20 (74.1) | 29 (87.9) | 49 (81.7) |
|  | Yes | 15 (37.5) | 15 (38.5) | 30 (38.0) | 7 (25.9) | 4 (12.1) | 11 (18.3) |
|  | N (*n* missing) | 40 (0) | 39 (0) | 79 (0) | 27 (13) | 33 (6) | 60 (19) |
| Attended an emergency dental appointment | No | 28 (70.0) | 31 (79.5) | 59 (74.7) | 25 (83.3) | 31 (86.1) | 56 (84.8) |
|  | Yes | 12 (30.0) | 8 (20.5) | 20 (25.3) | 5 (16.7) | 5 (13.9) | 10 (15.2) |
|  | N (*n* missing) | 40 (0) | 39 (0) | 79 (0) | 30 (10) | 36 (3) | 66 (13) |
| Attended an A&E department because of oral health | No | 37 (92.5) | 38 (97.4) | 75 (94.9) | 30 (100) | 36 (100) | 66 (100) |
|  | Yes | 3 (7.5) | 1 (2.6) | 4 (5.1) | 0 (0) | 0 (0) | 0 (0) |
|  | N (*n* missing) | 40 (0) | 39 (0) | 79 (0) | 30 (10) | 36 (3) | 66 (13) |
| Teeth brushing frequency | ≥2 a day | 19 (47.5) | 15 (39.5) | 34 (43.6) | 13 (48.1) | 17 (50.0) | 30 (49.2) |
|  | Once a day | 11 (27.5) | 12 (31.6) | 23 (29.5) | 6 (22.2) | 9 (26.5) | 15 (24.6) |
|  | <1 once a day | 10 (25.0) | 11 (28.9) | 21 (26.9) | 8 (29.6) | 8 (23.5) | 16 (26.2) |
|  | N (n missing) | 40 (0) | 38 (1) | 78 (1) | 27 (13) | 34 (5) | 61 (18) |
| Teeth brushing time | ≥2 minutes | 29 (76.3) | 33 (86.9) | 62 (81.6) | 21 (84) | 27 (84.4) | 48 (84.2) |
|  | 1 minute | 7 (18.4) | 4 (10.5) | 11 (14.5) | 3 (12.0) | 4 (12.5) | 7 (12.3) |
|  | <1 minute | 2 (5.3) | 1 (2.6) | 3 (3.9) | 1 (4.0) | 1 (3.1) | 2 (3.5) |
|  | N (*n* missing) | 38 (2) | 38 (1) | 76 (3) | 25 (15) | 32 (7) | 57 (22) |
| Cleaning between teeth frequency | At least once a day | 6 (15.0) | 3 (7.9) | 9 (11.5) | 5 (18.5) | 1 (3.0) | 6 (10.0) |
|  | 1-2 times per week | 3 (7.5) | 1 (2.6) | 4 (5.1) | 2 (7.4) | 9 (27.3) | 11 (18.3) |
|  | Monthly | 2 (5.0) | 3 (7.9) | 5 (6.4) | 2 (7.4) | 1 (3.0) | 3 (5.0) |
|  | Never | 29 (72.5) | 31 (81.6) | 60 (76.9) | 18 (66.7) | 22 (66.7) | 40 (66.7) |
|  | N (*n* missing) | 40 (0) | 38 (1) | 78 (1) | 27 (13) | 33 (6) | 60 (19) |
| Using fluoride mouthwash | No | 13 (32.5) | 16 (41.0) | 29 (36.7) | 11 (40.7) | 16 (45.7) | 27 (43.5) |
|  | Yes | 27 (67.5) | 23 (59.0) | 50 (63.3) | 16 (59.3) | 19 (54.3) | 35 (56.5) |
|  | N (*n* missing) | 40 (0) | 39 (0) | 79 (0) | 27 (13) | 35 (4) | 62 (17) |
|  |  |  |  |  |  |  |  |
| *Based on responses to the Brief Pain Inventory | |  |  |  |  |  |  |
| **Based on responses to the Manchester Orofacial Pain & Disability Scale. | | |  |  |  |  |  |
